# Questioning hagfish affinities of the enigmatic Devonian vertebrate *Palaeospondylus*

**DOI:** 10.1098/rsos.170214

**Published:** 2017-07-19

**Authors:** Zerina Johanson, Moya Smith, Sophie Sanchez, Tim Senden, Kate Trinajstic, Cathrin Pfaff

**Affiliations:** 1 Department of Earth Sciences, Natural History Museum, London, UK; 2 Tissue Engineering and Biophotonics, Dental Institute, King's College London, London, UK; 3 Department of Organismal Biology, Uppsala University, Uppsala, Sweden; 4 European Synchrotron Radiation Facility, Grenoble, France; 5 Department of Applied Mathematics, Research School of Physics and Engineering, Australian National University, Canberra, Australian Capital Territory 2601, Australia; 6 Environment and Agriculture, Curtin University, Kent Street, Bentley, Perth, Australia; 7 Department of Palaeontology, University of Vienna, Vienna, Austria

**Keywords:** *Palaeospondylus*, X-ray tomography, hagfish, Chondrichthyes, jawed vertebrates, chondrocranium

## Abstract

*Palaeospondylus gunni* Traquair, 1890 is an enigmatic Devonian vertebrate whose taxonomic affinities have been debated since it was first described. Most recently, *Palaeospondylus* has been identified as a stem-group hagfish (Myxinoidea). However, one character questioning this assignment is the presence of three semicircular canals in the otic region of the cartilaginous skull, a feature of jawed vertebrates. Additionally, new tomographic data reveal that the following characters of crown-group gnathostomes (chondrichthyans + osteichthyans) are present in *Palaeospondylus*: a longer telencephalic region of the braincase, separation of otic and occipital regions by the otico-occipital fissure, and vertebral centra. As well, a precerebral fontanelle and postorbital articulation of the palatoquadrate are characteristic of certain chondrichthyans. Similarities in the structure of the postorbital process to taxa such as *Pucapampella*, and possible presence of the ventral cranial fissure, both support a resolution of *Pa. gunni* as a stem chondrichthyan. The internally mineralized cartilaginous skeleton in *Palaeospondylus* may represent a stage in the loss of bone characteristic of the Chondrichthyes.

## 1. Introduction

*Palaeospondylus gunni* Traquair, 1890 is commonly found in the Middle Devonian Achanarras fish beds, Achanarras Quarry (Scotland), part of a deeper-water lake fauna including jawless fishes ([1–3], but see [4] for possible marine influence) and gnathostomes including placoderms, acanthodians and osteichthyans. The fishes occur throughout several beds, with *Palaeospondylus* co-occurring with the lungfish *Dipterus*, the acanthodian *Mesacanthus,* and the placoderm *Pterichthyodes* [5]. Previously, *Palaeospondylus* has been assigned to almost every major jawless and jawed vertebrate group and identified as both larval and adult [6–11]. Most recently, Hirasawa *et al*. [12] described similarities between *Palaeospondylus* and larvae of the extant hagfish *Eptatretus burgeri*, suggesting a hagfish affinity, and more particularly as a stem hagfish. However, new X-ray tomographic scans of *Palaeospondylus* provide important new details of cranial anatomy, particularly with respect to the otic capsule and vestibular system, allowing us to identify *Palaeospondylus* as a jawed vertebrate rather than a jawless hagfish. More specifically, crown-group gnathostome characteristics (elongate telencephalon of the braincase, vertebral centra) are present in *Palaeospondylus*, additionally the large L-shaped element on the lateral braincase is identified as a postorbital process, with the palatoquadrate articulating posteroventrally on this process. Along with a precerebral fontanelle, these suggest a chondrichthyan affinity for *Palaeospondylus*, with similarity to the stem chondrichthyans such as *Pucapampella* [13–15].

## 2. Material and methods

### 2.1. Specimens

Specimens of *Pa. gunni* examined are from the Middle Old Red Sandstone, Achanarras Quarry, Scotland, including NHMUK PV P.22392, P.59351, P.66582, P.59333, P.59645, P66582 (Department of Earth Sciences, Natural History Museum, London).

### 2.2. X-ray tomography

The *Palaeospondylus* chondrocranium was scanned from 10 micro slices by the ultrafine computed tomography (CT) scanner in the Department of Applied Mathematics, Australian National University, Australia and at beamline ID19, European Synchrotron Radiation Facility, France (see the electronic supplementary material for technical details). The high-resolution scans presented here all have a voxel size of 5 µm. A movie of the TIFF stack, from lateral to medial, is available at the Natural History Museum (NHM) Data Portal (see Data accessibility, below). Scans were three-dimensional volume rendered using Drishti v. 2.4 software (sf.anu.edu.au/Vizlab/drishti/).

### 2.3. Three-dimensional volumetric rendering

We analysed the synchrotron data with the software CT-Analyser (v. 1.14.4.1) and CTvox (v. 2.7.0) by Bruker/Skyscan. Working with different grey-values and transparency, CT-Analyser allowed us to distinguish the *Pa. gunni* specimen (P.66582) from the surrounding sediment. Details of the CTvox settings are found in the electronic supplementary material. Three-dimensional segmentation of internal structures was not possible.

### 2.4. Macrophotography

Specimens were photographed with a Canon EOS 1100D, and a Leica MZ microscope (Leica Application Suite 2.8.1), with images processed in Adobe Photoshop (CC 2014.2.2) to improve contrast.

## 3. Results

**Figure 1.**
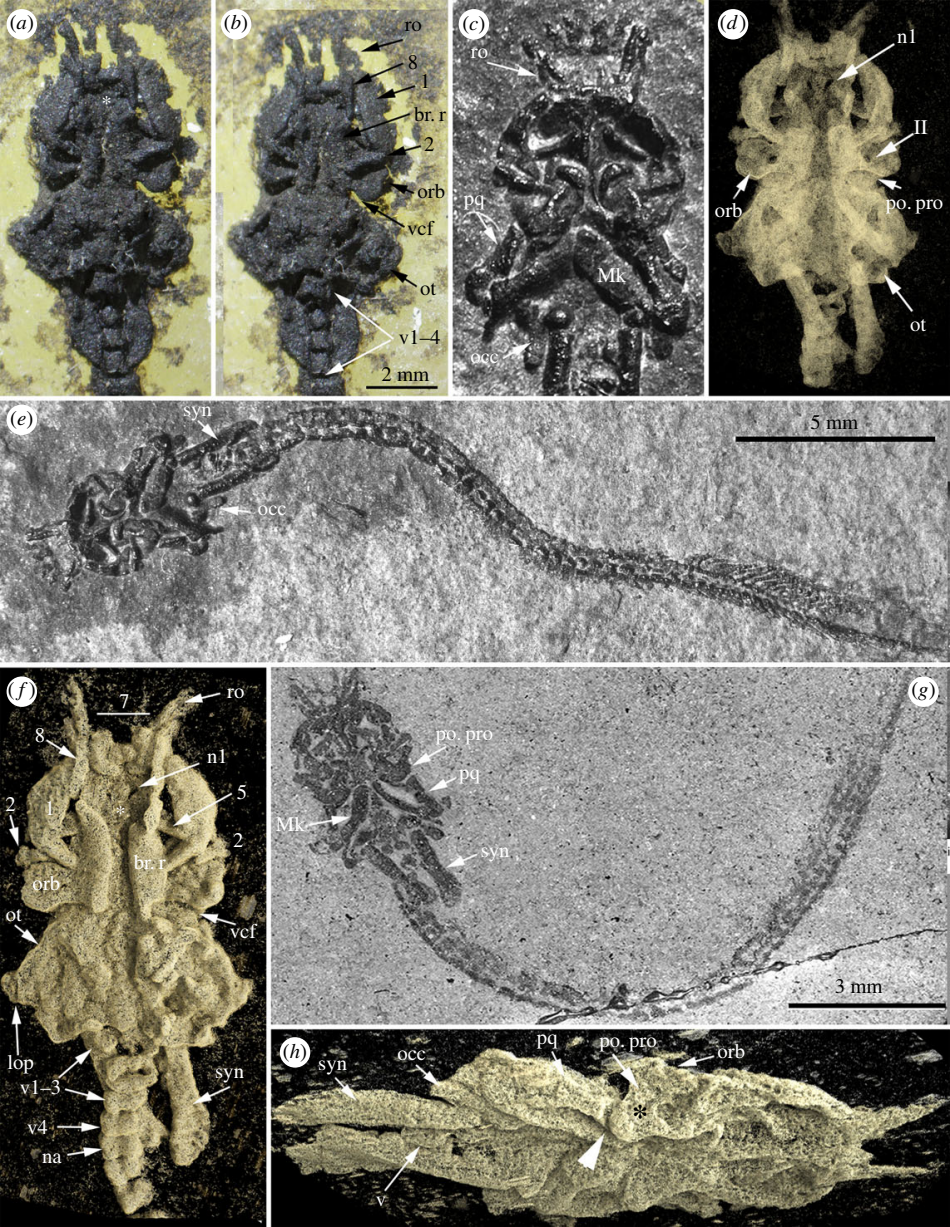
*Palaeospondylus gunni*, Achanarras Quarry (Devonian), Scotland. (*a,b*) NHMUK PVP22393, stereopair. Asterisk indicates position of the precerebral fontanelle; (*c,e*) NHMUK PVP59563, macrophotograph of ventral chondrocranium (*c*), and entire specimen (*e*); (*d,f,h*) NHMUK PVP66582, chondrocranium and anterior vertebrae including lateral elements (syn) forming synarcual anteriorly. Detailed mineralized tissue structure is resolved in the entire skeleton, round lacunae surrounded by interlacunar mineralization and dense perilacunar tissue. (*d*) Ventral, (*f*) dorsal and (*h*) ventrolateral views, three-dimensional volume rendered (Drishti). Asterisk in (*h*) indicates position of jugular canal, white arrowhead the posteroventral articulation of the palatoquadrate on the postorbital process; (*g*) ventral view of entire specimen, macrophotograph. 1–5, elements of the nasal capsule; 6–8, elements associated with rostral structure (see the electronic supplementary material, figure S1 for numbers not shown here); II, optic cranial nerve; br.r, braincase roof; lop, lateral otic process; Mk, Meckel's cartilage; n1, nerve foramen (medial supraopthalmic branches of trigeminal, facial cranial nerves); na, neural arch; occ, occipital; orb, orbit; po.pro, postorbital process; ot, otic capsule; pq, palatoquadrate; ro, rostral sensory structure; syn, lateral elements forming synarcual with anterior vertebral elements; tr, trabeculae; vcf, ventral cranial fissure; v, vertebral elements.

Individuals of *Pa. gunni* have a distinctive morphology, with a large chondrocranium, mandibular arch skeleton and extensive vertebral column with a well-developed caudal fin (figure 1*e,g*; [6]). More posterior branchial arches and paired appendages appear to be absent, even as imprints, as do unpaired dorsal and anal fins. The vertebral column comprises stout elements through most of its length but anteriorly displays an unusual series of vertebrae associated with two blade-like, posteriorly directed structures (figure 1; electronic supplementary material, figure S1). *Palaeospondylus* is normally preserved in dorsal (figure 1*a,b,d,f*) or ventral view (figure 1*e,g*); new X-ray tomographic data allow for a modified three-dimensional visualization in lateral view (figure 1*h*; [16]), allowing for a revised interpretation of the prominent L-shaped structure on the lateral face of the chondrocranium and the element articulating to the posteroventral margin of this structure. A more complete examination of *Palaeospondylus* morphology is provided in the electronic supplementary material; phylogenetically relevant characters, particularly with respect to the recent identification of *Palaeospondylus* as a hagfish, are described below.

### 3.1. Phylogenetically important characters of *Palaeospondylus*

**Figure 2.**
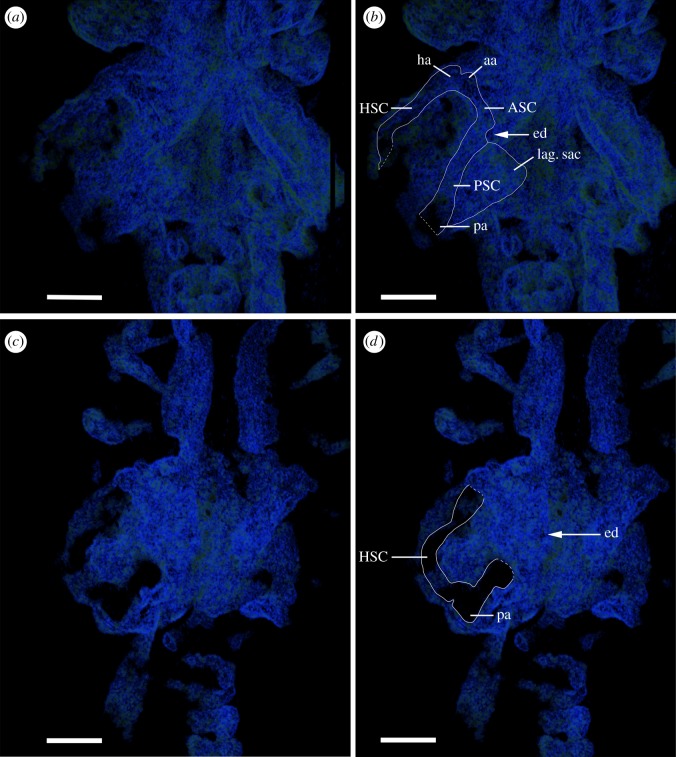
*Palaeospondylus gunni*, Achanarras Quarry (Devonian), Scotland. (*a,b*) NHMUK PVP P66582, ventral view of the otic region, volumetric rendered (CTvox), for better visualization of internal structures dorsal and ventral parts of the specimen are removed. (*c,d*) More ventral tilted ‘virtual’ plane through the specimen. ASC, anterior semicircular canal; PSC, posterior semicircular canal; HSC, horizontal semicircular canal; aa, anterior ampulla; ed, endolymphatic duct; pa, posterior ampulla; ha, horizontal ampulla; lag.sac, sacculo-lagenar sac.

The most recognizable parts of the *Pa. gunni* chondrocranium are the otic capsules at the posterior margin of the braincase (figure 1*a,b,d,f*; ot, figure 2*a,b*), which occupy almost one-third of the cranium. Internally, the otic region preserves three relatively wide semicircular canals (figure 2; ASC, anterior semicircular canal; PSC, posterior semicircular canal; HSC, horizontal semicircular canal) and the three associated ampullae (aa; anterior ampulla; pa, posterior ampulla; ha, horizontal ampulla; figure 2*b*), which are filled partially with sediment. Additionally, an endolymphatic duct between the ASC and PSC may be recognized, opening onto the dorsal surface of the braincase (figure 2*b,d*; ed, electronic supplementary material, figure S1*c*; ed?). A large sac-like region, the saccular or sacculo-lagenar sac, is present, below and medial to the ASC and PSC, shifted from its usual ventral position during post-mortem compression (figure 2*b*; lag.sac). Otoliths within this sac are absent.

Immediately posterior to the otic capsules and separate from these [16] are small, comma-shaped structures. Given their position relative to the otic capsules, and their similarity to comparable structures in some bony fishes ([17]: pls 24, 66), they are identified as the occipitals (figure 1*c,e,h*; occ). Separation of the occipitals implies the presence of a fissure between the otic and occipital regions. Additionally, a ventral cranial fissure may be present, separating the otic capsules from the more anterior ethmosphenoid region of the braincase (figure 1*a,b,f*; electronic supplementary material, figure S1*a*; vcf). At the posterior margin of the chondrocranium, paired foraminae are visible (electronic supplementary material, figure S1*b,d*; lda), an assumed entrance of the lateral dorsal aorta.

Anterior to the otic capsules on the lateral chondrocranium, is a large, L-shaped structure enclosing a foramen near its base (figure 1*h*, electronic supplementary material, figure S1*b,d*; asterisk). Dorsally, this structure is flat and is associated with a foramen for cranial nerve II (optic nerve) in the sidewall of the chondrocranium (figure 1*d*, electronic supplementary material, figure S1*a*, orb, II; [18,19]). This association, and location anterior to the otic capsule suggests that this is the floor of the orbit. Nerve II marks the anterior border of the diencephalon with the telencephalon, suggesting that this is relatively long in *Palaeospondylus*. The L-shaped structure beneath and behind the orbit is interpreted as the postorbital process. Two elements articulate with the postorbital process, forming a V-shaped structure ventrally (figure 1*c,e,g*); these are interpreted as the palatoquadrate and Meckel's cartilage, forming the mandibular arch. Generally, the hyomandibular (hyoid arch) articulates posterior to the ventral cranial fissure on a process associated with the otic region [20], suggesting that these structures anterior to the fissure are reasonably identified as the mandibular arch.

The chondrocranium anterior to the orbit is difficult to interpret, although this region must include the nasal capsules (see the electronic supplementary material). Medially, a pair of elongate elements forms the roof of the chondrocranium, but do not extend to the anterior margin of the chondrocranium. This leaves a substantial open area anteriorly [18], reminiscent of the precerebral fontanelle (figure 1*a,b,f*, br.r, electronic supplementary material, figure S1*a*, asterisk; [21]). Additional morphological features are described in the electronic supplementary material.

### 3.2. *Palaeospondylus* as a stem-group hagfish

Previously, *Palaeospondylus* was identified as either a member of the Cyclostomata or related to this group in some way [22–26], and most recently as a stem-group hagfish, based on similarities of *Palaeospondylus* to specific embryonic developmental stages of the hagfish *E. burgeri* [12]. However, our new data show that several structures have been misidentified in *Palaeospondylus*, including a cage-shaped nasal capsule, two pairs of dorsal longitudinal cartilage bars linked by commissures, and a velar bar and large lingual plates ventrally. In *E. burgeri* embryos, the pairs of longitudinal cartilages run anteroposteriorly along the chondrocranium, linked by transverse commissures and are continuous with the otic capsule posteriorly. In *Palaeospondylus*, the more medial of these putative longitudinal cartilages (forming the braincase roof as described above, figure 1*a,b*,*f*; br.r) were reconstructed as continuous with the anterior margin of the otic capsule [12, fig. 3*a,b*], but in fact, these elements are distinct from the otic region, marking the position of the possible ventral cranial fissure (figure 1*a,b,f*; vcf). Transverse commissures 1 and 2 are also incorrectly identified in *Palaeospondylus*, with the first being a distinct and separate element identified here as part of the nasal capsule (electronic supplementary material, figure S1*b*; 5). This element is entirely within the chondrocranium (although visible in external view; figure 1*f*), rather than being an external commissure [12, fig. 3*a*, comm.1]. Commissure 2 is also a separate element in *Palaeospondylus*, forming an anterior wall to the orbit (figure 1*a,b,f*; electronic supplementary material, figure S1*b*). In both cases, these elements are not continuous or contiguous with other cartilaginous parts of the braincase.

Following Bulman [18], Hirasawa *et al*. [12, fig. 3*b,d*] identified a series of ventral elements in *Palaeospondylus* (e.g. figure 1*c,e,g*) as the velar bar and lingual bar, comparable to *E. burgeri*. The velar bar was reconstructed as a single continuous element [12], but it is composed of two opposing structures (figure 1*g*; pq, Mk); moreover, as described above, these were identified as the palatoquadrate articulating with Meckel's cartilage, with the palatoquadrate articulating to the ventral postorbital process. Hirasawa *et al*. [12, fig. 3*b*] reconstructed what is clearly the separate palatoquadrate (e.g. figure 1*c,g*, electronic supplementary material, figure S1*b*) as incorporated into the anterior margin of the otic capsule (also [18]).

One of the two elements forming the putative lingual bar [12] was identified above as the occipital (also [16]), while the second element articulates to these occipitals, and is associated with the unusual anterior region of the vertebral column comprising narrow, rounded centra and more posterior vertebral elements. Centra are absent from the hagfish vertebral column ([27]; present only in certain crown gnathostome taxa [28]), and we provide a novel interpretation of this region, that it represents the region of the anterior vertebral column, the synarcual (electronic supplementary material). The presence of centra within the synarcual of *Palaeospondylus* is reminiscent of the synarcual in batoid chondrichthyans [29,30].

Hirasawa *et al*. [12] also focused on the crown-like structure at the anterior chondrocranium, identifying this as a nasal capsule, comparable to embryos of *E. burgeri*. This comparison depends on identification of anterior and posterior transverse bars connecting the separate longitudinal bars of the crown to form a cage-like structure, as in the *E. burgeri* nasal capsule (stage 60 [12]: fig. 3a). However, the transverse anterior bar does not exist ([6,16,21,22], contra [31]; figure 1; electronic supplementary material, figure S1*b*), and there is no particular resemblance to the nasal capsule, which we identify as being paired and positioned more posteriorly to the rostral crown-like structure; instead this region is identified as a rostrum at the anterior chondrocranium and potentially an innervated sensory organ (electronic supplementary material). Cumulatively, these misidentifications [12] strongly argue against *Palaeospondylus* being a hagfish (also recently rejected by Janvier & Sansom [32]), moreover, characters discussed above, in particular the vestibular system with its three semicircular canals within the otic capsule, suggest that *Palaeospondylus* is a jawed vertebrate, with several characters shared with crown-group gnathostomes.

## 4. Discussion

The characters described above establish *Palaeospondylus* as a jawed vertebrate, contrary to recent suggestions by Hirasawa *et al*. [12]. Apart from the mineralized vertebrae (e.g. centra), the most convincing new character arguing against a myxinoid and agnathan relationship is the third semicircular canal in the otic region of *Palaeospondylus* and associated ampullae. This is characteristic of jawed vertebrates (e.g. [33]), but not extant agnathans (one semicircular canal [12]) or extinct taxa (anterior and posterior canals) lacking the horizontal canal [34–37]. However, the isolation of posterior semicircular canals from the other canals in modern elasmobranchs [38,39] is not seen in *Palaeospondylus.* Additionally, a potential endolymphatic duct can be identified via an opening into the duct (figure 2*b–d*; electronic supplementary material, figure S1*c*), a character of extinct and extant craniates [39].

Crown-group gnathostome characters in *Palaeospondylus* also include the elongate telencephalon, vertebral centra and possibly the ventral cranial fissure [40,41], although the former may also be characteristic of the closely related stem gnathostome *Janusiscus* [20]. The precerebral fontanelle [21,42] and the articulation of the palatoquadrate on the large L-shaped postorbital process on the lateral chondrocranium [13,14,42,43] indicate a closer relationship with chondrichthyans. Certain Palaeozoic stem chondrichthyans have a postorbital process that includes a ventral contribution from the lateral commissure (processes of the otic region) to enclose the jugular vein [14]. If this were the case in *Palaeospondylus*, the postorbital process would cross onto the otic capsule and run across the ventral cranial fissure. However, in the stem chondrichthyan *Pucapampella*, it is the postorbital process that extends ventrally to enclose the jugular canal (also in other taxa such as *Cobelodus*, *Tamiobatis* and *Orthacanthus* [43]), more comparable to *Palaeospondylus*. *Pucapampella* also possesses an otico-occipital fissure, which is lost in more derived chondrichthyans [44]. Hence characters present in *Palaeospondylus* are also present in stem chondrichthyans.

## 5. Conclusion

*Palaeospondylus gunni* has been a perplexing vertebrate fossil since Traquair first described it in 1890; here X-ray tomography provides new data and morphological characters demonstrating that *Palaeospondylus* is a jawed vertebrate. Our interpretation of key jawed vertebrate characters in *Palaeospondylus* include three semicircular canals with a horizontal canal prominent in the vestibular system, the synarcual, and jointed elements of the mandibular arch (palatoquadrate, Meckel's cartilage). Moreover, multiple crown-group gnathostome characters are present (otico-occipital fissure (but also in ptyctodont placoderms [45])), elongate telencephalon, and vertebral centra. Characters that associate *Palaeospondylus* with chondrichthyans are a precerebral fontanelle, foramina for lateral dorsal aorta in the chondrocranium, and the articulation of the palatoquadrate to the ventral postorbital process.

This new analysis of *Palaeospondylus* strongly suggests that an assignment to the myxinoid (hagfish) stem group is based on misinterpretation of various characters [12]. However, the absence/non-preservation of teeth, scales and fins continues to be problematic in determination of *Palaeospondylus* as a jawed vertebrate. Nevertheless, a number of acanthodian taxa, resolved as stem chondrichthyans [40,42], also lack teeth [46]. As well, Miller [31] described maceration of the lungfish *Lepidosiren*, resulting in loss of the fins, girdles and branchial arches, comparable to post-mortem degradation potentially responsible for the absence of these elements in *Palaeospondylus*.

Also problematic with regards to a chondrichthyan association is the composition of the *Palaeospondylus* cartilaginous skeleton that includes hypertrophied chondrocyte lacunae surrounded by mineralized matrix, previously interpreted as representing an early stage in endochondral bone development [10,47], a type of bone found in bony fishes (Osteichthyes) [48]. The presence of hypertrophied cells in chondrichthyan cartilage is disputed [49,50], with mineralization characteristically only in the perichondral tesserae [51]. Nevertheless, chondrichthyans are characterized by loss of bone [48,52]; *Palaeospondylus* also lacks bone and instead manifests an entirely mineralized cartilage in the endoskeleton, with matrix mineralization between and around the cell lacunae (interlacunar, perilacunar), clearly illustrated in details of tissue structure (figure 1*f,h*). The fact that this mineralized tissue can be observed, and not perichondral bone, which normally forms in the connective tissue surrounding the cartilage, implies that the latter is indeed absent. As a stem chondrichthyan, *Palaeospondylus* may mark the loss of perichondral bone, as well as dermal bone, as part of the overall loss of bone within chondrichthyans, including acanthodians [48].

These novel characters and newly evaluated ones allow a more precise relationship of *Palaeospondylus* to be proposed, that can be challenged with new data, or new imaging techniques directed towards these hypotheses.

## Supplementary Material

Electronic Supplementary material - text

## Supplementary Material

ESM Figure S1 Palaeospondylus gunni, Achanarras Quarry (Devonian), Scotland.

## Supplementary Material

ESM Figure S2 Palaeospondylus gunni, Achanarras Quarry (Devonian), Scotland.

## Supplementary Material

ESM Figure S3 Palaeospondylus gunni, Achanarras Quarry (Devonian), Scotland.

## Supplementary Material

ESM Figure S4 Palaeospondylus gunni, Achanarras Quarry (Devonian), Scotland.

## Data accessibility

Data files, including movie through individual slices, can be found at the NHM Data Portal (http://data.nhm.ac.uk/dataset/palaeospondylus).
