# Supplementary material for: Questioning hagfish affinities of the enigmatic Devonian vertebrate Palaeospondylus
Source: R Soc Open Sci. 2017 Jul 19;4(7):170214. doi: 10.1098/rsos.170214 (PMC5541543; doi:10.1098/rsos.170214)
Supplement: Electronic Supplementary material - text [file rsos170214supp1.pdf]

Electronic Supplementary Material

Questioning hagfish affinities of the enigmatic Devonian vertebrate *Palaeospondylus gunni* Traquair, 1890

Zerina Johanson[1]*, Moya Smith[1,2,] Sophie Sanchez[3,4], Tim Senden[5], Kate Trinajstic[6], Cathrin Pfaff[7]

[1] Department of Earth Sciences, Natural History Museum, London, UK

[2] Tissue Engineering and Biophotonics, Dental Institute, King's College London, London, UK

[3] Department of Organismal Biology, Uppsala University, Uppsala, Sweden

[4] European Synchrotron Radiation Facility, Grenoble, France

[5] Department of Applied Mathematics, Research School of Physics and Engineering, Australian National University, Canberra, ACT 2601, Australia

[6] Environment and Agriculture, Curtin University, Kent Street, Bentley, Perth, Australia

[7] Department of Palaeontology, University of Vienna, Vienna, Austria

Chondrocranium

One feature of the *Palaeospondylus* specimens that may not have been appreciated previously is the substantial three-dimensionality of the chondrocranium (figure 1A, B, stereopair). Historically, NHMUK PV P22393 was prepared and removed from the surrounding rock matrix with hydrochloric acid [18], but it was judged too fragile for CT-scanning. Various features of the chondrocranium and postcranial skeleton are described below.

Postorbital process and mandibular arch. As mentioned in the main text, an L-shaped structure is present on the lateral face of the chondrocranium, enclosing a foramen near its base (figure 1H, electronic supplementary material, figures S1B, D, S4; po.pro, asterisk). Additionally, in dorsal view, the L-shaped structure forms a strongly concave surface (figure 1A, B, D, F, orb), with the sidewall of the braincase medial to this surface being pierced by a laterally oriented foramen (figure 1D, electronic supplementary material, figure S1A, II). Given the relative position of this surface, with the more rostral braincase morphology described below being suggestive of nasal capsules, we identify this concave surface as the floor of the orbit and the foramen for nerve II. Because nerve II is associated with the diencephalon region of the brain [40], we can determine that *Palaeospondylus* possessed a long telencephalon region.

In lateral view, an elongate structure articulates to the posteroventral margin of the L-shaped structure (figure 1G, H, electronic supplementary material, figure S1B, pq). At the distal margin of this elongate structure and seen in ventral view, a second elongate and thin element articulates (figure 1C, G, electronic supplementary material, figure S1B, Mk), together forming a V-shaped structure. In *Palaeospondylus gunni*, the articulated V-shaped unit has previously been identified as the hyomandibular arch (epihyal, ceratohyal), and the L-shaped structure as the palatoquadrate (figure 1H; [16]). Thomson et al. [16] reconstructed the hyomandibular as articulating to the braincase just posterodorsal to this palatoquadrate, but our new scans show that the 'hyomandibular' articulates directly to the 'palatoquadrate' (figure 1H, white arrowhead). However, the hyomandibular does not normally articulate with the

Deleted: 2

Deleted: 2

Deleted: 2

palatoquadrate, but on the otic capsule or transverse processes associated with the otic capsule ([20]: Suppl. fig. 9, grey arrows); in more basal placoderms such as *Brindabellaspis* and *Macropetalichthys*, the hyomandibular articulates anterior to the otic capsule, but also ventral or anterior to the orbit, not posteriorly [20, 42, 53-55].

The location of the L-shaped structure on the braincase and its relationship to the orbit suggest that it is better identified as the postorbital process (figure 1H, electronic supplementary material, figure S1B), rather than the transverse otic process *per se* (lateral commissure [20]). The L-shaped structure is pierced by a foramen suggested to be for the jugular canal (figure 1H, electronic supplementary material, figure S1B, asterisk). These observations have implications for the V-shaped unit articulating to the rear of the postorbital process: among jawed vertebrates, the palatoquadrate articulates with the postorbital process in chondrichthyans such as the stem chondrichthyans *Acanthodes* [42, 54], *Doliodus* [15] and *Cobelodus* [43] and among crown group chondrichthyans, the Hexanchiformes [14].

Meckel's cartilage: Meckel's cartilage is identified as the independent element articulating with the palatoquadrate and oriented ventromedially towards its opposite, forming a v-shaped structure in ventral view (figure 1C, G, Electronic Supplementary Material, figures S1B, 3; Mk). Together, the palatoquadrate and Meckel's cartilage (figure 1G, electronic supplementary material, figure S1B) form the mandibular arch. Neither teeth nor any dentition are preserved, nor more posterior branchial arches, the latter believed to be unmineralised, or removed post-mortem, comparable to observations made for the lungfish

Deleted: 2

Deleted: 2

Deleted: 2

Deleted: S2B

Lepidosiren [31]. There is no transverse otic process visible on the lateral otic capsule, so it is suggested the hyomandibular articulated to the otic capsule, as in chondrichthyans.

Anterior chondrocranium: The anterior chondrocranium is unusual, with multiple elements that are difficult to interpret and identify (figure 1A-D, F; electronic supplementary material, figure S1A, B). The largest of these is broadly semicircular, forming the lateral margins of the anterior chondrocranium (figure 1A, B, D, F, electronic supplementary material, figure S1B; 1). Medial to these are elongate elements that appear continuous with the crown-like rostral structure described further below (figure 1A, B; F, 8); posteromedially, a pair of structures extends posteriorly to the anterior margin of the otic capsules (figure 1A, B, F; br.r). These elements represent the chondrocranium roof, and appear separated in the midline, only extending anteriorly to the ventral end of element 8, leaving a substantial open space in the anterior braincase, identified in the main text as the precerebral fontanelle (figure 1A, B, F, electronic supplementary material, figure S1A, asterisk). Just anterior to the postorbital process/orbit floor is a pair of small, elongate elements (figure 1F; 2), which on the three-dimensionally preserved specimen form a vertical anterior wall to the orbit (figure 1A, B; 2). These can all be considered more external parts of the braincase. Internally and seen in ventral view, there are three additional elements, including a small medially directed structure and two on either side of this, anteriorly and posteriorly (electronic supplementary material figure, S1B; 3-5).

Deleted: S2A

Deleted: S2B

Deleted: S2A

Deleted: S2B

Given our identification of the otic and orbital regions, this anterior area is best interpreted as the nasal region (figure 1A, B, F; electronic supplementary material, figure S1B), with internal elements 3 and 4 representing the outer parts of the nasal capsule and the medial element (5) potentially providing support for a nasal mucosa. A pair of small foramina is present medially between elements 5 and 8 and visible in dorsal view (figure 1F, n1), believed to be for a pair of nerves associated with the rostralmost region, described below. These foramina are also visible in ventral view, located on either side of the posteriorly directed process of the teardrop-shaped structure at the anterior margin of the chondrocranium (electronic supplementary material, figure S1B; 6). In dorsal view, the foramina are also just posterior to a set of cartilages at the anterior margin of the chondrocranium (figure 1F; 7). These include a pair of small round elements medially, with a pair of semicircular cartilages laterally. Elements 6 and 7 are unusual structures for the anterior margin of a braincase, so we suggest they are also associated with the crown-shaped rostral structure, perhaps forming a base and attachment points for components of the rostral structure. Element 8 runs on either side of element 7 in dorsal view to frame precerebral fontanelle associated with the small foramina, described above.

Rostral structure (sensory): A crown-shaped series of thin, elongate structures extend from the anterior braincase margin (figure 1A-G, electronic supplementary material figure S1B, D; ro), associated with the unusual structures at the internal and external anterior margins of the chondrocranium, mentioned above (figure 1F; 7, electronic supplementary material, figure S1B;

Deleted: S2B

Deleted: S2B

Deleted: 2

6). Additionally, two sets of foramina are believed to be for nerves extending into this rostral region. The first is the small midline pair described above (n1), while the second is larger, and more laterally positioned. The latter extends from the anterior part of the nasal capsules (electronic supplementary material, figure S1A; n2), where the anterior margin appears pierced by a triangular notch, marking the position of a groove that continues rostrally, presumably towards the rostral structure. The identity of these notches/openings is discussed further below.

Postcranial skeleton

The vertebral column is well-developed, with the anteriormost elements represented by simple, thin circular centra (figure 1F, electronic supplementary material, figure S2C; v1-3) and more posterior vertebrae with neural arches and their bases articulating to the centra (electronic supplementary material, figure S2A, B; white asterisk). Posterior to the first three circular centra, these arches are small and low (figure 1F; v4 and more posterior 2-3 vertebrae; electronic supplementary material figure S2C). Elongate structures run alongside these centra and articulate with the occipital region of the braincase, previously identified as cranial ribs, described below as mineralisation associated with a modified vertebral, fused structure known as the synarcual (figure 1E-H, syn; [29, 30]).

Still more posteriorly, the neural arches become larger, rectangular and block-shaped (electronic supplementary material, figure S2A, C, D; na). Haemal arches are absent or unmineralised anteriorly (acid-prepared specimen, electronic supplementary material, figure S2A, D), and for most of

Deleted: S2A

Deleted: organ

Deleted: S1C

Deleted: S1A

Deleted: S1C

Deleted: but

Deleted: S1A

Deleted: S1A

the vertebral column. At some point, the column can become twisted ([6]); electronic supplementary material, figure S2D; point marked by X), providing a comparison of the axial skeleton in dorsolateral and ventral views. Ventrally, the vertebral column appears open (also figure 1E, G), suggesting the ventral parts of the centra are unmineralised [electronic supplementary material, figure S2D, asterisk; [6]), as discussed further below. Mineralised haemal arches are present closer to the caudal fin (electronic supplementary material, figure S2D; haem).

Deleted: S1D

Deleted: S1D

Deleted: S1D

Discussion

Just anterior to the precerebral fontanelle is a rostral structure, suggested to be supported by several cartilages at the anterior margin of the chondrocranium (elements 6-8; figure 1F; electronic supplementary material, figure S1B). Elongate cartilaginous elements comprising the rostrum have been described in other taxa, for example the dogshark [17, 55]. Moreover, the rostral structure in *Palaeospondylus* is positioned close to two pairs of openings in the braincase suggested to be nerve foramina. Two cranial nerves that extend rostrally include the facial and trigeminal; for example, in a range of teleosts, supraorbital and infraorbital branches of these nerves supply the sensory barbels at the front of the head [56, 57], the facial nerve in particular being associated with taste buds on the barbels [58]. We suggest that the more medial supraopthalmic branches of these nerves ran through the small medial openings in the *Palaeospondylus* chondrocranium (figure 1D, F, electronic supplementary material, figure S1B; n1), while the more laterally directed branches (maxillary, mandibular) passed through the dorsolateral

Deleted: 2

Deleted: S2B

openings at the anterior margin of the nasal capsules (electronic supplementary material, figure S1A; n2). Olfactory nerves do not normally supply sensory tentacles or barbels. Given the position of the crown-shaped rostral structure at the anterior margin of the chondrocranium, and the suggested association with at least two sets of cranial nerves, we suggest this represents a large sensory organ in *Palaeospondylus*, with the individual parts being comparable to the barbels that characterise several extant fish taxa, including those with a cartilaginous core [57].

Deleted: S2A

The position of the orbit and cranial nerve II (figure 1D, electronic supplementary material, figure S1A) also establish the position of the diencephalon, and the size of the more anterior telencephalic region of the braincase. In *Palaeospondylus*, the telencephalon would have been long, around half the length of the chondrocranium. Dupret et al. [40: fig. 3] mapped the length of the telencephalon onto a jawed vertebrate cladogram, demonstrating that a marked increase in length occurred in the crown gnathostomes. However, more recently described taxa such as the stem gnathostome *Janusiscus* may also show a longer telencephalon (based on the position of the postorbital process and the hypophyseal fossa, also related to the position of the diencephalon [30: extended data figure 7K].

Deleted: S2A

A pair of large foramina are located at the posterior edge of the chondrocranium, which we suggested were possibly for the passage of the lateral dorsal aorta through the cartilaginous braincase, these foramina being considered a character of the chondrichthyan total group ([41]: Fig. 9), although they are also present in the placoderm *Brindabellaspis* ([51]: Fig. 15B).

Deleted: . This character is important phylogenetically, with

The comma-shaped structures interpreted as occipitals are distinct from otic capsules [16], suggesting the presence of the otico-occipital fissure, which characterises crown-group gnathostomes [30: fig. 3], but is also present in the unusual braincase of the ptyctodont placoderms [45]. The elongate elements articulating with the occipital (figure 1E-H; syn) were previously identified as the cranial ribs (e.g., [8, 16]), a character aligned with lungfish. However, identification of *Palaeospondylus* as a lungfish has been criticised [7, 9] and as described above, these ‘ribs’ extend posteriorly in association with the anterior three circular centra, and three additional vertebrae with reduced neural arches, as part of the synarcual of the anterior vertebral column. The more posterior vertebral elements include centra with neural arches with higher, larger, blockier spines. Modification of the anterior vertebral column occurs in a wide variety of jawed vertebrates (synarcual in placoderms, chondrichthyans; Weberian apparatus, teleost fishes), and we suggest the elongate elements (‘ribs’) represent modified and fused ventral vertebral elements, similar to the ventral part of the synarcual recently illustrated in a variety of placoderms and the chondrichthyans *Chimera monstrosa* and *Dasyatis americana* [30: figs 2, 4, 6, 7]; along with the circular centra and following (posterior, modified) vertebrae with low neural arches, these represent the synarcual. As noted, modification and fusion of anterior vertebral elements is characteristic of multiple jawed vertebrate taxa including placoderms and chondrichthyans (batoids and holocephalans) [29, 30, 59, 60], although why the anterior three centra are completely mineralised as part of the synarcual, but more posterior centra (associated with the neural arches) are unmineralised ventrally, along with haemal arches (until near the caudal

fin) is uncertain. Nevertheless, the presence of centra in the postcranial skeleton is a character of crown group gnathostomes [28], and associated with the batoid but not holocephalan synarcual [29, 30].

Based on the new synchrotron data, the mandibular arch has been identified in *Palaeospondylus*; however, more posterior branchial arches are not preserved. The hyomandibular was said to attach to a lateral process on the otic capsule above, but no other attachment surfaces are present, suggesting the branchial arches would have been positioned posterior to the braincase, rather than beneath it (e.g., holocephalans, amphibians). This is more similar to stem chondrichthyans [61], a characteristic that now can also be suggested for *Palaeospondylus*.

The *Palaeospondylus* skeleton is composed of mineralised cartilage, but bone is absent (reviewed in [10, 47]). The chondrichthyan skeleton is also entirely cartilage, although patterns of cartilage mineralization differ between *Palaeospondylus* and chondrichthyans. In chondrichthyans, superficial (perichondral tesserae) mineralization dominates (e.g., [50]), while in *Palaeospondylus* mineralization was throughout all the cartilage, both within the matrix (interlacunar) and encircling large lacunae (perilacunar), these suggested to be related to hypertrophied (enlarged) cells within the cartilage [10, 47]. This hypertrophy and specific localised mineralization were suggested to be normal initial stages in endochondral bone formation, a characteristic of osteichthyan fishes. Some cells in chondrichthyan cartilage do become hypertrophied [49], but the matrix is not mineralised, this being restricted to the perichondral tesserae [51]. This may preclude the assignment of *Palaeospondylus* to the total group Chondrichthyes. However, as outlined in

Deleted: if crown group Gnathostomata (Chondrichthyes + Osteichthyes) is characterized by endochondral bone, retained in Osteichthyes, but lost in Chondrichthyes

the main text, *Palaeospondylus* as a stem-group chondrichthyan may represent an early evolutionary stage in the loss of bone in the group [48], particularly perichondral bone, which is present in some acanthodians (also stem-group chondrichthyans [e,g, 62]).

Material and Methods

Synchrotron data

Palaeospondylus gunni was imaged using the powerful X-ray beam at the beamline ID19 from the European Synchrotron Radiation Facility (Grenoble, France) [63]. A monochromatic beam was produced through the wiggler opening of 35mm and reflected by the double Si111 Bragg monochromator. The resulting energy was of 40 keV. A FreLON 2k14 CCD detector [64] with a 10 μm thick Gadox scintillator were used to scan the sample with a voxel size of 7.46 μm. In order to use phase contrast, the sample was fixed at the distance of 1000 mm from the detector. 1999 projections were taken over 360°. The time of exposure was of 1 s. A single phase retrieval approach [65] (modified from the algorithm of Paganin et al. [66]) was used to reconstruct the data.

References

51 Zhu M, Yu X, Ahlberg PE, Choo B, Lu J, Qiao T, Qu Q, Zhao W, Jia L, Blom, H, Zhu Y. 2013. A Silurian placoderm with osteichthyan-like marginal jaw bones. *Nature* **502**,188–193. DOI:10.138/nature12617

52 Stensiö EA. 1969. Elasmobranchiomorphi. Placodermata. Arthrodires. *In:* J. Piveteau (ed.) *Traité de Paléontologie* **4(2),**71-692. Paris: Masson S.A.

53 Young GC. 1980. A new early Devonian placoderm from New South Wales, Australia, with a discussion of placoderm phylogeny. *Palaeont. Abt. A* **167**,10-76.

54 Brazeau MD, de Winter V. 2015. The hyoid arch and braincase anatomy of Acanthodes support chondrichthyan affinity of 'acanthodians', *Proc. Roy. Soc. B* **282**, DOI:10.1098/rspb.2015.2210

55 Reynolds SH. 1897. The Vertebrate Skeleton. Cambridge Biological Series, Cambridge University Press, Cambridge. pp 588.

56 Saxena PK. 1966. Cranial nerves of the common catfish, *Clarias batrachus* (LINN.). *Jpn. J. Ichthyol.* **14**, 91-98.

57 Fox H. 1999. Barbels and barbel-like tentacular structures in sub-mammalian vertebrates: a review. *Hydrobiologia* **403**,153–193. DOI:10.1023/A:1003778125517.

58 LeClair EE, Topczewski J. 2010. Development and regeneration of the zebrafish maxillary barbel: A novel study system for vertebrate tissue growth and repair. *PLoS ONE* **5(1)**: e8737. DOI:10.1371/journal.pone.0008737

59 Johanson Z, Boisvert C, Maksimenko A, Currie P, Trinajstic K. 2015. Development of the synarcual in the Elephant Sharks (Holocephali; Chondrichthyes): Implications for vertebral formation and fusion. *PLoS One* **10(9)**, e0135138. DOI: 10.1371/journal.pone.0135138.

60 VanBuren C, Evans D. 2016. Evolution and function of anterior cervical vertebral fusion in tetrapods. *Biol. Rev*. DOI: 10.1111/brv.12245

61 Pradel, A., Didier D, Casane D, Tafforeau, P, Maisey, J. G. 2013. Holocephalan embryo provides new information on the evolution of the glossopharyngeal nerve, metotic fissure and parachordal plate in gnathostomes. *PLoS One* **8(6)**: e66988. DOI:10.1371/journal.pone.0066988

62 Burrow CJ, Trinajstic K, Long JA. 2011. First acanthodian from the Upper Devonian (Frasnian) Gogo Formation, Western Australia. *Hist. Biol*. **24**: 349-357

63 Tafforeau P, et al. 2006. Applications of X-ray synchrotron microtomography for non-destructive 3D studies of paleontological specimens. *Applied Physics A - Materials Science & Processing* **83**, 195-202.

64 Labiche J-C, Mathon O, Pascarelli S, Newton MA, Guilera Ferre G, Curfs C, Vaughan G, Homs A, Fernandez Carreiras D. 2007. The fast readout low noise camera as a versatile X-ray detector for time resolved dispersive extended X-ray absorption fine structure and diffraction studies

of dynamic problems in materials science, chemistry, and catalysis. *Review of Scientific Instruments* **78**, 091301-091311.

65 Sanchez S, Ahlberg PE, Trinajstic K, Mirone A, Tafforeau P. 2012. Three dimensional synchrotron virtual paleohistology: a new insight into the world of fossil bone microstructures. *Microscopy and Microanalysis* **18**, 1095-1105.

66 Paganin D, Mayo SC, Gureyev TE, Miller PR, Wilkins SW. 2002. Simultaneous phase and amplitude extraction from a single defocused image of a homogeneous object. *Journal of Microscopy* **206**, 33-40.

Figure Captions

Figure S1. *Palaeospondylus gunni*, Achanarras Quarry (Devonian), Scotland. A, NHMUK PVPP59333, 3D rendered (Drishti). Asterisk indicates position of the precerebral fontanelle; B, NHMUKPVP66582, chondrocranium, ventral view, 3D rendered (Drishti). Asterisk indicates position of jugular canal, white arrowhead the posteroventral articulation of the palatoquadrate to Meckel's cartilage. C, NHMUK PVP22393, macrophotograph of dorsal chondrocranium indicating possible position of left endolymphatic duct. D, line drawing of B, ventral view of chondrocranium, black asterisk indicates position of jugular canal. **Abbreviations**: As in Figures 1, 2.

Figure S2 *Palaeospondylus gunni*, Achanarras Quarry (Devonian), Scotland. A, B, NHMUK PVP22393, macrophotograph of anterior axial skeleton in lateral view. B, closeup showing articulation of neural arch bases to the centra

(asterisk). C, D. NHMUK PVP61428, macrophotographs showing postcranial skeleton in dorsoventral (C) and dorsal and ventral (D) views. In D, X marks position where vertebral column rotates postmortem from dorsal to ventral view. Asterisks indicate incompletely mineralized centra (unmineralised ventrally). **Abbreviations**: As in Figures 1, 2, also cent, centrum, haem, haemal arch.

Figure S3 *Palaeospondylus gunni*, Achanarras Quarry (Devonian), Scotland. Line drawing, ventral view of chondrocranium. **Abbreviations**: As in Figures 1, 2, S1, S2.

Figure S4 *Palaeospondylus gunni*, Achanarras Quarry (Devonian), Scotland. Line drawing, ventrolateral view of chondrocranium. Black asterisk indicates position of jugular canal. **Abbreviations**: As in Figures 1, 2, S1, S2.
