# Supplementary figures and images for: Questioning hagfish affinities of the enigmatic Devonian vertebrate Palaeospondylus
Source: R Soc Open Sci. 2017 Jul 19;4(7):170214. doi: 10.1098/rsos.170214 (PMC5541543; doi:10.1098/rsos.170214)

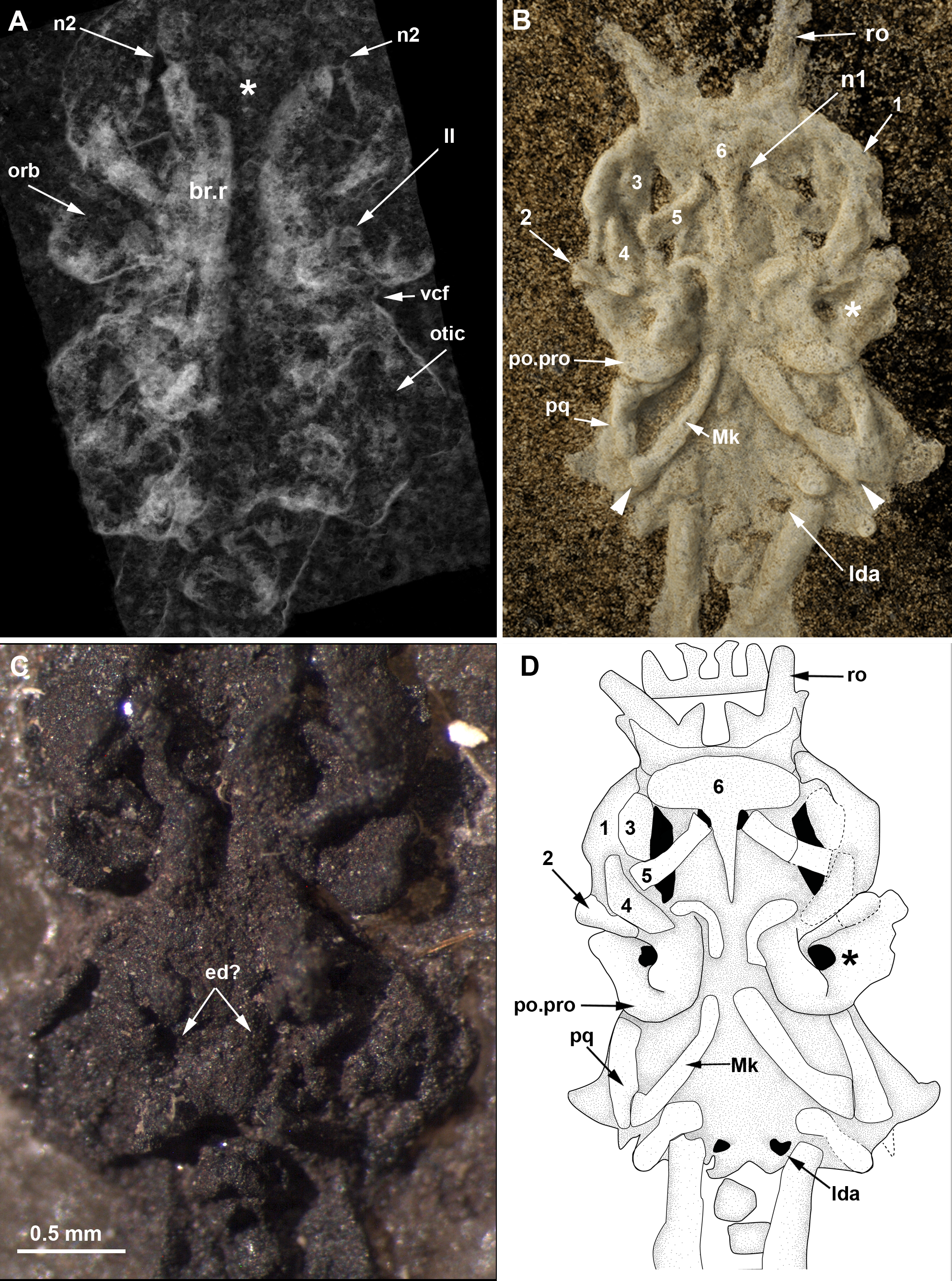

Supplement: ESM Figure S1 Palaeospondylus gunni, Achanarras Quarry (Devonian), Scotland. [file rsos170214supp2.tif]

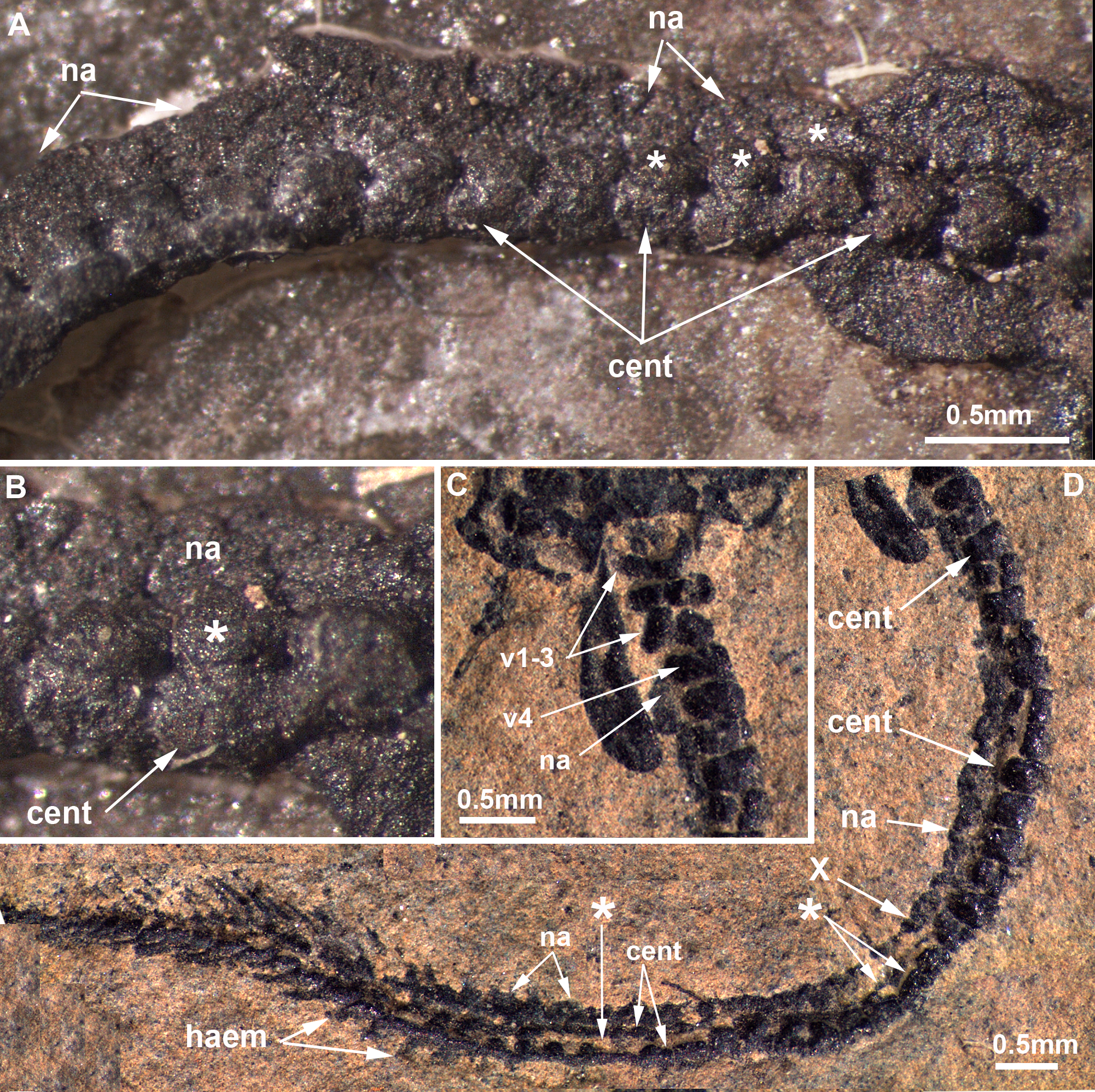

Supplement: ESM Figure S2 Palaeospondylus gunni, Achanarras Quarry (Devonian), Scotland. [file rsos170214supp3.tif]

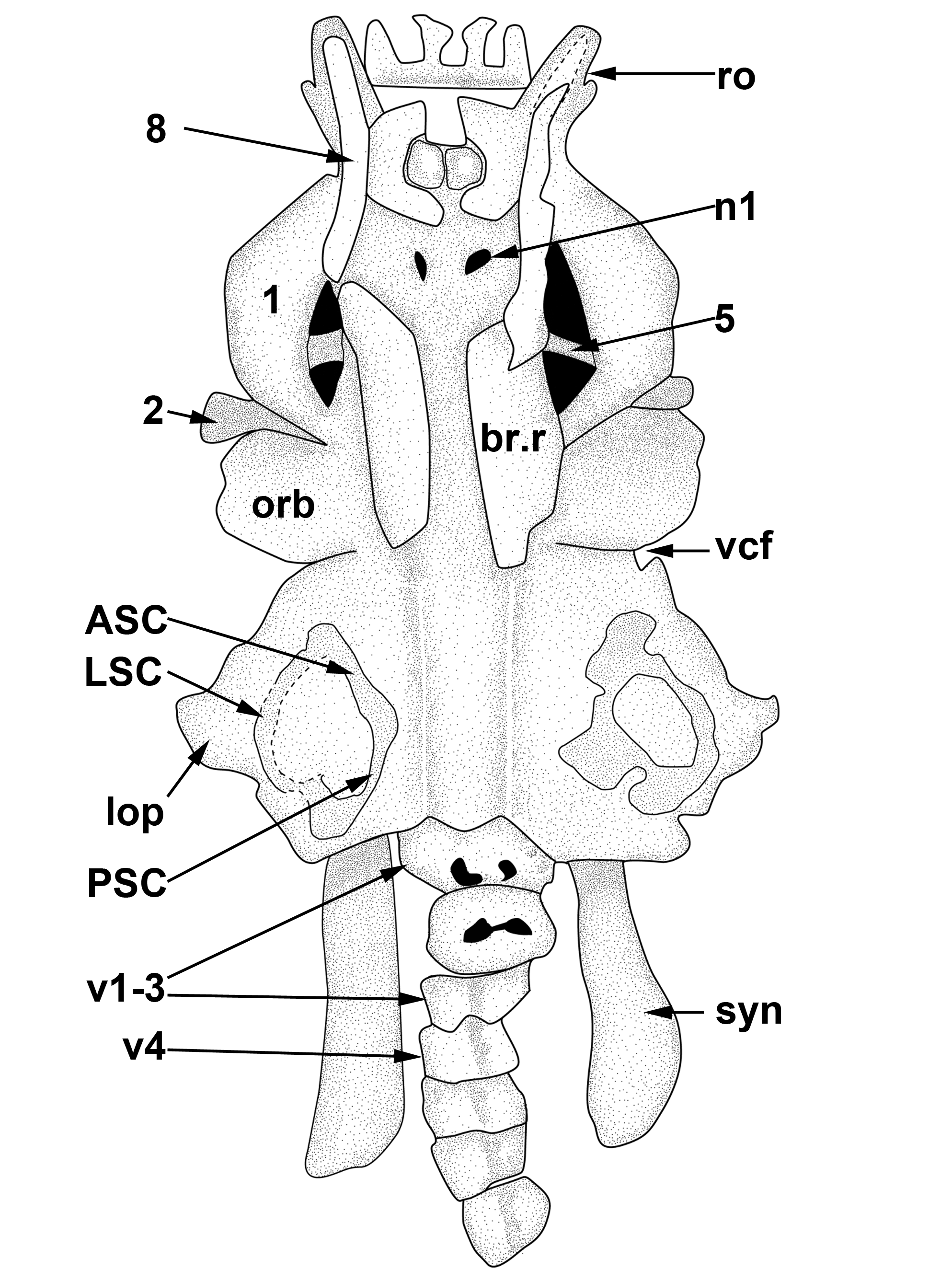

Supplement: ESM Figure S3 Palaeospondylus gunni, Achanarras Quarry (Devonian), Scotland. [file rsos170214supp4.tif]

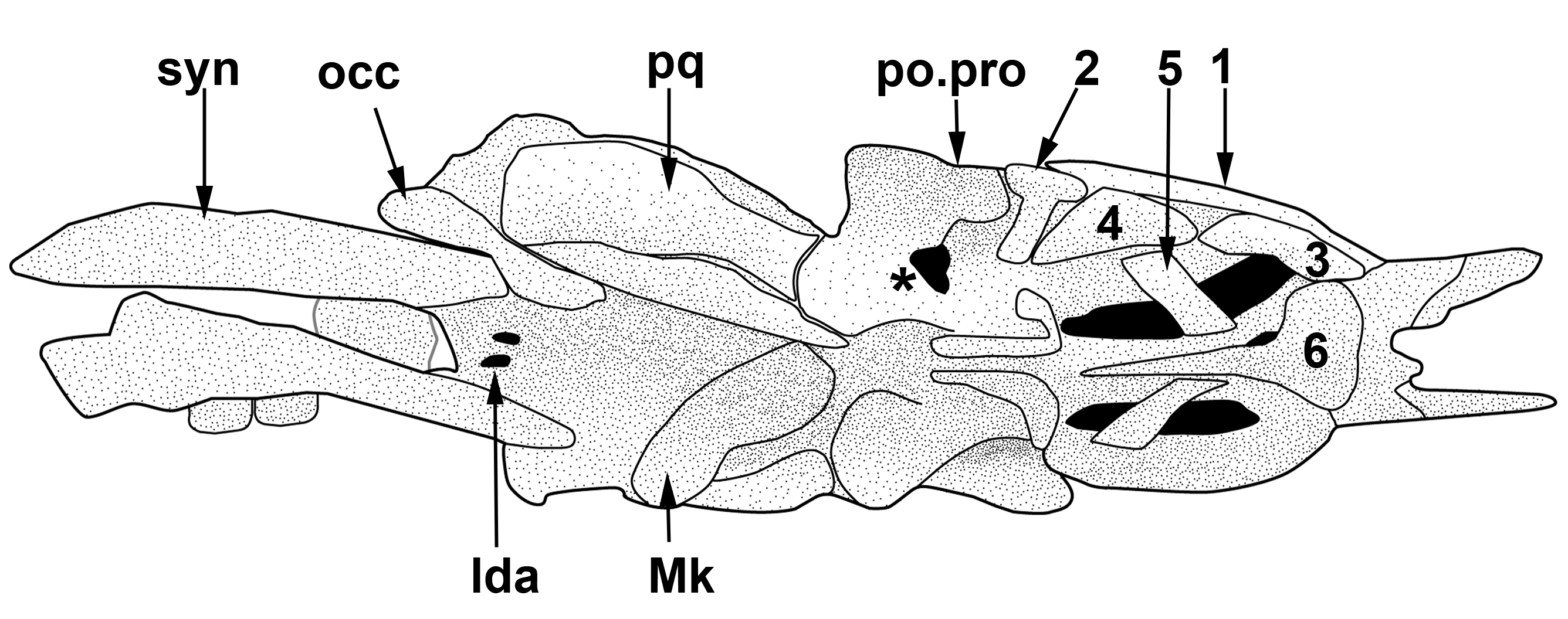

Supplement: ESM Figure S4 Palaeospondylus gunni, Achanarras Quarry (Devonian), Scotland. [file rsos170214supp5.tif]
